# A novel deletion in 2q24.1q24.2 in a girl with mental retardation and generalized hypotonia: a case report

**DOI:** 10.1186/1755-8166-5-1

**Published:** 2012-01-03

**Authors:** Orazio Palumbo, Pietro Palumbo, Teresa Palladino, Raffaella Stallone, Leopoldo Zelante, Massimo Carella

**Affiliations:** 1 Medical Genetics Unit, IRCCS Casa Sollievo della Sofferenza, San Giovanni Rotondo (FG), Italy

**Keywords:** mental retardation, 2q24.1q24.2, array comparative genomic hybridization

## Abstract

**Background:**

Chromosomal imbalances, recognized as the major cause of mental retardation, are often due to submicroscopic deletions or duplications not evidenced by conventional cytogenetic methods. To date, interstitial deletion of long arm of chromosome 2 have been reported for more than 100 cases, although studies reporting small interstitial deletions involving the 2q24.1q24.2 region are rare. With the widespread clinical use of comparative genomic hybridization chromosomal microarray technology, several cryptic chromosome imbalances have outlined new genotype-phenotype correlations and isolated a number of distinctive clinical conditions.

**Results:**

here we report on a girl with mental retardation and generalized hypotonia. A genome-wide screen for copy number variations (CNVs) using single nucleotide polymorphisms (SNPs) array revealed a 7.5 Mb interstitial deletion of chromosome region 2q24.1q24.2 encompassing 59 genes, which was absent in parents. The gene content analysis of the deleted region and review of the literature revealed the presence of some genes that may be indicated as good candidate in generating the main clinical features of the patient.

**Discussion:**

the present case represents a further patient described in the literature with an interstitial deletion of chromosome 2q24.1q24.2. Our patient shares some clinical features with the previously reported patients carriers of overlapping 2q24 deletion. Although more cases are needed to delineate the full-blown phenotype of 2q24.1q24.2 deletion syndrome, published data and present observation suggest that hemizygosity of this region results in a clinically recognizable phenotype. Considering these clinical and cytogenetic similarities, we suggest the existence of an emerging syndrome associated to 2q24.1q24.2 region.

## Background

Conventional cytogenetic analysis have identified more than 100 individuals with constitutional deletion within 2q. In particular, over 70 patients with a terminal deletion and over 30 with an interstitial deletion have already been reported [1,2]. Most of the examined patients presented a mental retardation varying between severe and profound, hypotonia and dysmorphic features. Phenotype variations are likely to be due to differences in the size and location of the segmental aneuploidy. The most frequent interstitial deletion involves the cytogenetic bands 2q31q33 and corresponds to a specific phenotype [3,4]. Only few cases showed overlapping deletions, although of quite different extensions, and most of the reported cases with 2q interstitial deletion have been detected with standard cytogenetic techniques showing poor definition of breakpoints. Thus, genotype-phenotype correlations in cases with a 2q deletion not involving 2q31q33 bands are more difficult. With the advent of Array based comparative genomic hybridization (array-CGH), the detection rate of submicroscopic chromosomal abnormalities have been improved considerably. To our best knowledge, studies reporting small interstitial deletions involving or partial overlapping the 2q24.1q24.2 region are rare. Takatsuki et al. [5] reported a patient with a deletion of 2q24.2q24.3 with delayed growth, mental retardation, generalized hypotonia, myoclonic seizures, and peculiar dysmorphic features, affected by severe pulmonary emphysema. Subsequently, Magri et al. [6] reported a patient with severe mental retardation, muscular hypotonia and characteristic dysmorphic features, who carried a de novo 5.3 Mb deletion in 2q24.2q24.3.

Moreover, in ECARUCA database was reported a patient, ID 4547, carrying a deletion in 2q24.2 who presented mental retardation, hypotonia and joint laxity.

Here we describe a patient carrying a de novo interstitial deletion of chromosome 2q24.1q24.2. Size and breakpoint of the deletion were determinate through SNP-Array analysis. A comparison of the clinical features in the individual reported here to those of previously reported individuals, suggests that haploinsufficiency of some genes encompassed by the deletion could possibly be related to mental retardation and hypotonia in patients with deletion of 2q24.1q24.2.

Finally, we further demonstrate the importance of the use of array-CGH technology in clinical setting to perform genotype-phenotype correlation.

## Case presentation

The Caucasian patient is the second child of a 42-years-old mother and 43-years-old father. The parents were healthy and non-consanguineous. Facial appearance was normal. She was born after an uneventful pregnancy. There was no family history of children born with congenital disease, intellectual disability, autism, seizures, neurologic disorders, metabolic disorder, recurrent pregnancy loss or infertility. Birth weight was 2700 g (50°C), height was 46,5 cm (< 3°C) and head circumference was 31 cm (< 3°C). Clinical examination revealed the presence of bilaterally hip dislocation. At 5 months of life the patient showed generalized hypotonia that required hospitalization. Karyotype and genetic test for Wolf syndrome, Rett syndrome, Spinal Muscular Atrophy, and Prader-Willi syndrome resulted normal. She started to speech some words when she was 3 years old. At that time her weight was 12,6 Kg (25°C), height was 93 cm (50°C), and cranial circumference 48 cm (10°C). She began to show a marked bruxism at age 4 years. Several instrumental examinations were performed in order to exclude the presence of other congenital malformation. Audiometric evaluation did not show hearing impairment, cerebral magnetic resonance imaging (MRI) did not reveal any alteration, cardiovascular, respiratory and abdominal examinations were normal.

Metabolic studies including plasma amino acids, lactate, glucose, transferring isoelectric-focusing, urine organic acids, urine oligosaccharides were all normal.

## Results

Conventional karyotyping by GTG banding at 550 bands of resolution revealed an apparently normal female karyotype of 46,XX.

**Figure 1:**
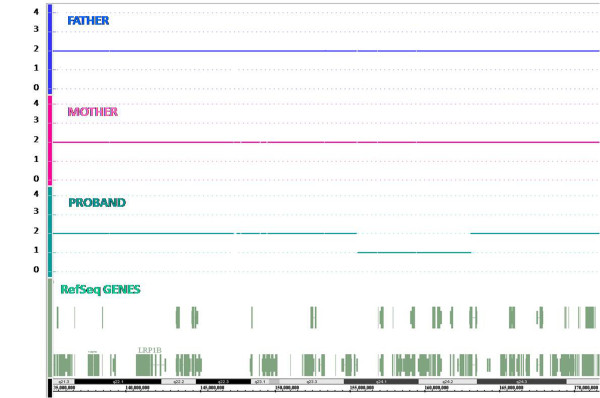
**Results of Affymetrix 250 K NspI array**. Copy number state of each probe is drawn along chromosome 2 from 135.000.000 to 170.000.000 bp. The upper panel represents the copy number state of the father, the middle panel the mother and the lower panel the proband. Values of Y-axis indicate the inferred copy number according to probe intensities.

Microarrays analysis was carried out using the GeneChip Human Mapping 250 K NspI Array. This analysis detected a 7.5 Mb deletion in the long arm of chromosome 2, from band 2q24.1 to band 2q24.2 (NCBI genome build 36). The breakpoint in band 2q24.1 was located between the last present probe SNP_A-2022259 (155.413.315 bp) and the first deleted probe SNP_A-4194091 (155.526.470 bp), while the breakpoint in band 2q24.2 was located between the last deleted probe SNP_A-4234249 (163.058.894 bp) and the first present probe SNP_A-1954343 (163.101.007 bp) (Figure 1).

The deletion was confirmed by a second experiment of SNPs array using the GeneChip Human Mapping 250 K StyI Array. Array analysis of the parents showed no abnormalities demonstrating a "de novo" origin of the deletion in the patient (Figure 1).

## Discussion

We present a case with a 7.5 Mb *de novo *deletion of chromosome 2q24.1q24.2 encompassing 59 genes detected by SNP array. Clinical features include mental retardation, generalized hypotonia, marked bruxism and bilaterally hip dislocation. A comparison of the clinical features observed in our patient with the previously reported cases identified by array comparative genomic hybridization, that clearly overlap the current case, shows several common features including mental retardation and hypotonia. This deletion overlaps the deletion recently reported by Takatsuki et al. and two other patients: one described by Magri et al. and one reported in ECARUCA database (ID 4547). Takatsuki et al. described a patient with delayed growth, mental retardation, generalized hypotonia, myoclonic seizures, and peculiar dysmorphic features, affected by severe pulmonary emphysema. In this paper, the *ITGB6 *gene was indicated as responsible of the pulmonary phenotype observed in the patient described. According to these authors, the master genes responsible for myoclonic seizures are *SCN1A *and *SCN2A *(two genes located outside the boundaries of our deletion), while we are in agreement with Magri et al. about the role of ITGB6. In fact, despite the evidence that this gene was deleted in our patient and in the patient reported by Magri et al., no pulmonary diseases were recorded in his case history. Although it would seem to be no evidence indicating a strong relationship between haploinsufficiency of *ITGB6 *and pulmonary dysfunction, to indicate a defective penetrance or, alternatively, to suggest that additional genes are requested in causing this peculiar clinical sign, we suggest a clinical surveillance against pulmonary diseases in patients with 2q deletions encompassing *ITGB6*.

**Figure 2:**
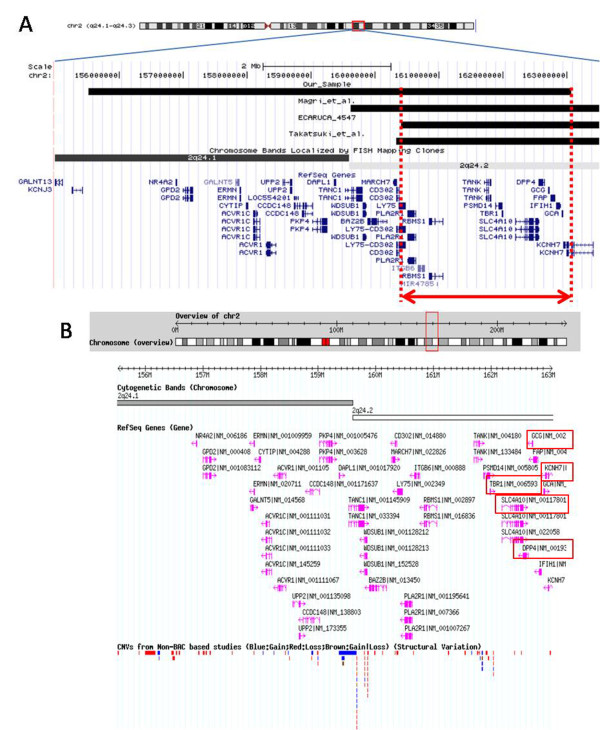
**Screenshot of the deleted region**. (A) Gene content of the deleted regions based on UCSC Genome Browser. The black boxes represent our case plus the patients reported by Magri et al., in ECARUCA database (ID 4547) and by Takatsuki et al. The red arrow shows the critical region shared by the patients (B) Copy number variations present in the deleted region based on Database of Human genomic variants.

The other two patients, one described by Magri et al. and one reported in ECARUCA database (ID 4547), presented non-specific abnormalities such as mental retardation, prenatal growth retardation and sever hypotonia. The deleted region shared by all the patients (from 160.408.000 bp to 163.058.894 bp; hg 18) (Figure 2A) contains 15 genes among which at least 5 candidate genes (*SLC4A10, TBR1, KCNH7, DPP4, GCG*) could be related to clinical features presented by our patient. Copy number variants have not been detected in normal individuals (Database of Human genomic variants, http://projects.tcag.ca/variation accessed June 2011) for these five genes (Figure 2B).

Solute carrier family 4, sodium bicarbonate transporter, member 10 (*SLC4A10*, MIM: 605556) belongs to a small family of sodium-coupled bicarbonate transporters (NCBTs) that regulate the intracellular pH of neurons, the secretion of bicarbonate ions across the choroid plexus, and the pH of the brain extracellular fluid. Disruption of sodium bicarbonate transporter *SLC4A10 *it has also been associated with mental retardation and epilepsy [7].

T-box, brain, 1 (*TBR1*, MIM: 604616) is a member of a conserved family of genes that share a common DNA-binding domain, the T-box. T-box genes encode transcription factors involved in the regulation of developmental processes. A similar protein has been disrupted in mice and shown to be critical for early cortical development, and causes loss of projection neurons in the olfactory bulbs and olfactory cortex [8].

Potassium voltage-gated channel, subfamily H (eag-related), member 7 (*KCNH7*, MIM: 608169) encodes a member of the potassium channel, voltage-gated, subfamily H. This family of channels are characterized by their anomalous gating behavior, with inactivation kinetics being faster than activation kinetics and with recovery from inactivation being faster than deactivation. Their diverse functions include regulating neurotransmitter release, heart rate, insulin secretion, neuronal excitability, epithelial electrolyte transport, smooth muscle contraction, and cell volume [9].

Since these genes have an important role in the brain, their haploinsufficiency could possibly be related to mental retardation and developmental delay in the patients.

Mental retardation and hypotonia have been associated in some cases with alteration in the carbohydrate metabolism [6]. In particular, in the region 2q24 encompassed by the deletion, there are two genes (*DPP4*, MIM: 102720; *GCG*, MIM: 138030) involved in carbohydrate metabolism.

Obviously, all suggested clinical features postulated to relate to loss of one copy of a particular candidate gene located inside the deleted region remain to be elucidated by further gene expression studies either on experimental in vivo animal models or on diagnostic material.

In conclusion, we suggest that deletions of chromosome 2q24.1q24.2 could be associated to a specific phenotype, although a clear genotype-phenotype correlation cannot be drawn yet, due to the low number of patients described and the large size of the deletions. Future genotype-phenotype comparison studies with more cases, along with gene expression studies, are needed to define whether a one-gene haploinsufficiency or more putative genes of the suggested critical regions could be associated with each major clinical features of 2q24.1q24.2 deletion phenotype.

## Materials and methods

### SNP Array analysis

High molecular weight genomic DNA was extracted from peripheral blood lymphocytes of the patient and both his parents, according EZ1 Robot procedure (Qiagen, Valencia, CA). DNA concentration was determined with NanoDrop ND-1000 spectrophotometer and software (NanoDrop Technologies, Berlin, Germany), while genome wide single nucleotide polymorphism (SNP) array analysis was carried out using the GeneChip Human Mapping 250 K NspI (Affymetrix, Santa Clara, CA, USA), which contains 25-mer oligonucleotide representing a total of 262.264 SNPs with an average resolution of ~12 kb. An amount of 250 ng DNA was processed according to the manufacturer's instructions. Briefly, total genomic DNA was digested with a restriction enzyme (NspI), ligated to an appropriate adapter for the enzyme, and subjected to PCR amplification using a single primer. After digestion with DNase I, the PCR products were labeled with a biotinylated nucleotide analogue, using terminal deoxynucleotidyl transferase and hybridized to the microarray. The wash and staining steps were performed using the Fluidic Station 450 and finally the array was scanned with the GeneChip Scanner 7 G using Command Console Software (Affymetrix, Santa Clara, CA, USA). Quality control (QC) and copy number analysis was performed using Genotyping Console Software 4.0 (Affymetrix, Santa Clara, CA, USA). Briefly: i) the raw data files (.CEL) were normalized using the default options; ii) an unpaired analysis was perfomed using as baseline the 270 HapMap samples in order to obtain Copy numbers value from .CEL files while the amplificated and/or deleted region were detected using a standard Hidden Markov Model (HMM) method.

Copy number changes identified in the samples were visualized by using the UCSC Genome Browser website http://genome.ucsc.edu and also compared to the Database of Genomic Variants http://projects.tcag.ca/variation to exclude copy number changes considered as benign variants. The DECIPHER https://decipher.sanger.ac.uk/ and ECARUCA http://umcecaruca01.extern.umcn.nl:8080/ecaruca/ecaruca.jsp databases were expedient as resources to aid genotype-phenotype correlation.

## Consent

Written informed consent was obtained from the patient for publication of this Case report and any accompanying images. A copy of the written consent is available for review by the Editor-in-Chief of this journal.

## Competing interests

The authors declare that they have no competing interests.

## Authors' contributions

OP analyzed and interpreted the array CGH data, wrote the paper. PP analyzed the databases and literature. TP and RS performed the experimental procedure. LZ initiated the clinical and laboratory investigations of the patient, and provided the phenotype information. MC coordinated the study and co-wrote the manuscript. All authors read and approved the final manuscript.
